# Pre-interventional renal artery calcification and survival after transcatheter aortic valve implantation

**DOI:** 10.1007/s10554-024-03295-5

**Published:** 2024-12-07

**Authors:** N. E. Winkler, J. Galantay, M. Hebeisen, T. G. Donati, J. Stehli, A. M. Kasel, H. Alkadhi, T. D. L. Nguyen-Kim, F. C. Tanner

**Affiliations:** 1 Department of Cardiology, University Heart Center, University Hospital Zurich and University of Zurich, Zurich, Switzerland; 2 Department of Biostatistics, Epidemiology, Biostatistics and Prevention Institute, University of Zurich, Zurich, Switzerland; 3 Diagnostic and Interventional Radiology, University Hospital Zurich, University of Zurich, Zurich, Switzerland; 4 Institute of Diagnostic and Interventional Radiology, Stadtspital Zurich, Zurich, Switzerland; 5 Department of Cardiology, University Heart Center, Raemistrasse 100, Zurich, CH-8091 Switzerland

**Keywords:** Aortic stenosis, Renal, Transthoracic echocardiography, Computed tomography, Prognosis, Mortality

## Abstract

The prognostic significance of renal artery calcification (RAC) is unknown in patients with severe aortic stenosis (AS) eligible for transcatheter aortic valve implantation (TAVI). RAC can be assessed by computed tomography (CT) performed during pre-interventional planning for TAVI. This study aimed at investigating the utility of RAC for predicting survival after TAVI. In this longitudinal cohort study, RAC volume was measured by CT in 268 consecutive patients with severe AS undergoing TAVI. Association of RAC with mortality was assessed using Cox regression analysis. RAC was evaluated as a binary parameter and in a supplementary analysis as a logarithmically transformed continuous variable. Over a median follow-up time of 9.6 years, 237 (88.4%) patients died, with 174 (73.4%) deaths attributable to a cardiovascular cause. RAC was highly prevalent (*N* = 150 (86.2%)) among patients suffering cardiovascular death. Competing risk cumulative incidence curves revealed a higher occurrence of cardiovascular death in patients with RAC (P-value = 0.008), while this was not the case for non-cardiovascular death (P-value = 0.71). RAC was independently associated with cardiovascular death (HR 1.61 [95% CI: 1.01–2.57]; *P* = 0.047) after adjustment for age, sex, cardiovascular risk factors, impaired renal function, and aortic valve calcification. The presence or absence of RAC rather than its volume was important in all the analyses. RAC is a strong and independent predictor of cardiovascular death in patients with severe AS undergoing TAVI. Given its favourable properties for event prediction, RAC may be considered valuable for prognostic assessment of TAVI patients.

**Graphical Abstract:**

**Figure:**
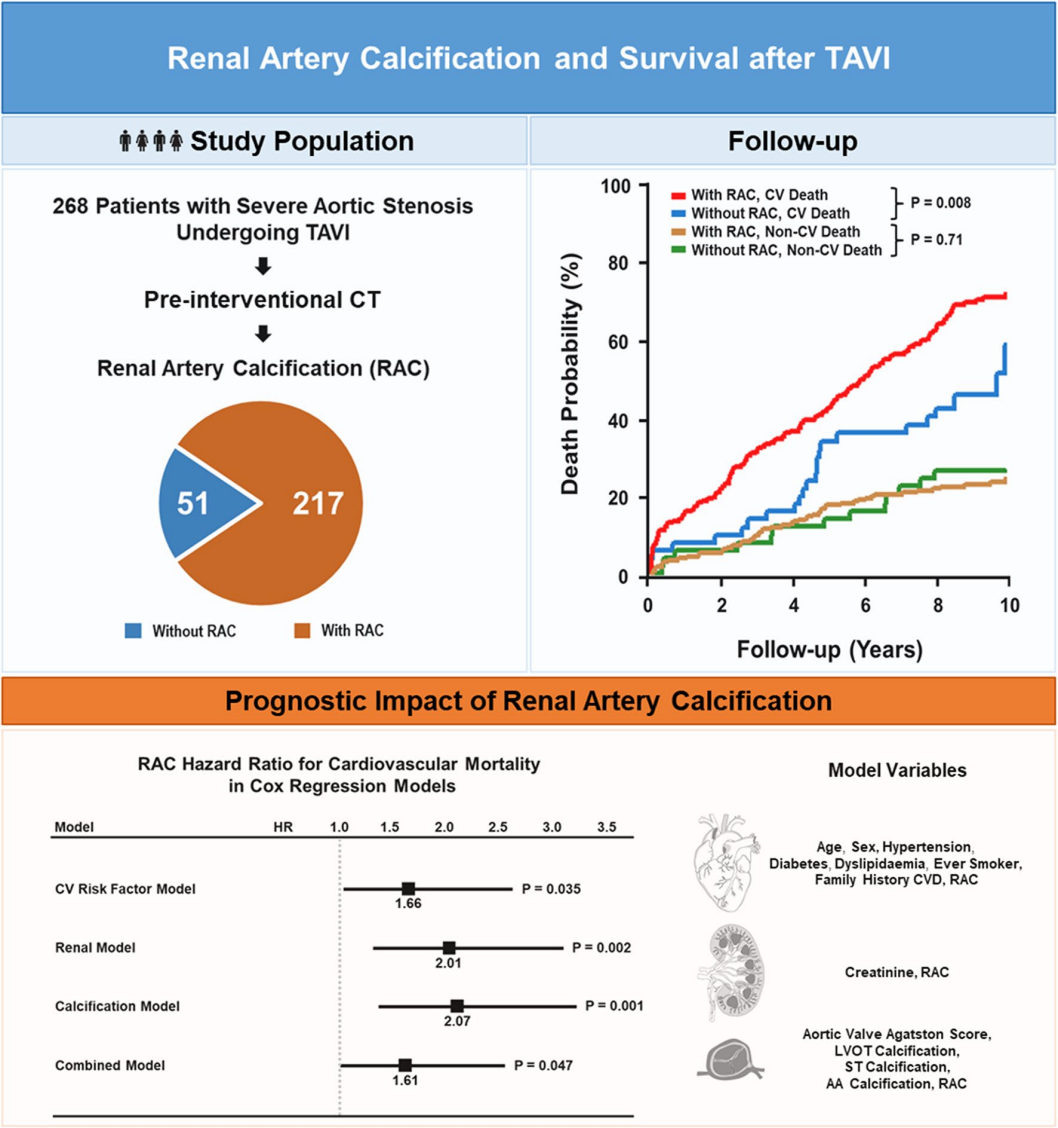
Occurence and prognostic implications of pre-interventional renal artery calcification after transcatheter aortic valve implantation. CV, cardiovascular; CVD, cardiovascular disease; LVOT, left ventricular outflow tract; CT, computed tomography; RAC, renal artery calcification; ST, sinotubular; AA, ascending aorta; TAVI, transcatheter aortic valve implantation

**Supplementary Information:**

The online version contains supplementary material available at 10.1007/s10554-024-03295-5.

## Lay Summary

This pioneering study investigated the association between renal artery calcification (RAC) and cardiovascular mortality in 268 consecutive patients with severe aortic stenosis (AS) eligible for transcatheter aortic valve implantation (TAVI). The findings indicated a robust association between RAC and cardiovascular death, independent of traditional risk factors such as age, sex, cardiovascular risk factors, renal function, and aortic valve calcification. Notably, the presence of RAC, rather than its volume, was significant. The readily available imaging marker RAC was investigated for the first time in this context and emerged as a strong and independent predictor of cardiovascular death in severe AS patients undergoing TAVI. Conversely, the absence of RAC reduced the risk of cardiovascular death by approximately 40%. In conclusion, this study suggests that RAC has significant prognostic value in assessing TAVI patients, underscoring its importance in predicting cardiovascular outcomes in these patients.

## Introduction

Degenerative aortic stenosis (AS) is the most frequent valvular heart disease in Western societies and exhibits an increasing incidence due to ageing of the population [1]. Transcatheter aortic valve implantation (TAVI) improves survival of patients with severe AS with or without left ventricular (LV) dysfunction [2–5]. While transthoracic echocardiography (TTE) serves as the primary cardiac imaging tool for diagnosing AS and grading its severity [1, 6], computed tomography (CT) plays an important role in the pre-interventional assessment of aortic valve morphology and calcification as well as in the evaluation of vascular access routes required for planning of TAVI [1, 7–9].

Atherosclerosis is a slowly progressive disease characterised by a complex pathogenesis including vascular inflammation and cell death, eventually resulting in calcification across multiple vascular sites [10]. Depending on the anatomical location and specific pathological alterations, atherosclerotic vascular disease may lead to diverse clinical manifestations associated with impaired outcome [11–13]. Nevertheless, very little is known about renal artery calcification (RAC) and its potential impact on outcome. RAC has been linked to various cardiovascular risk factors such as age, male sex, and arterial hypertension [14, 15]. A study performed in 4450 healthy individuals revealed that RAC was associated with an increased all-cause mortality risk independent of classic cardiovascular risk factors and atherosclerosis in other vascular beds [15].

Acute and chronic renal dysfunction has been recognised as an indicator of adverse outcome after TAVI [16–19]. However, it remains unknown whether RAC is associated with increased mortality after such an intervention. The renal arteries are contained in the three-dimensional data volume generated in CT scans required for pre-interventional planning of the TAVI procedure. This study aimed to investigate the utility of RAC in predicting survival after TAVI in patients with severe AS undergoing routine pre-interventional CT.

## Methods

### Study population

This longitudinal cohort study included consecutive patients with severe AS (aortic valve area (AVA) ≤ 1 cm^2^ or indexed AVA ≤ 0.6 cm^2^/m^2^ and/or mean transaortic pressure gradient (MTPG) ≥ 40 mmHg) identified from the prospective TAVI Registry of the University Heart Center Zurich, Switzerland. Long-term follow-up patients underwent TAVI following the Heart Team’s decision between July 2008 and January 2014. Patients were included when all the following conditions were fulfilled: (1) approval by local ethics committee and informed consent available, (2) complete TTE within six months before TAVI, (3) and pre-interventional CT. Patients younger than 18 years of age, exhibiting concomitant moderate or severe valvular heart disease, or having undergone valve-in-valve procedure or kidney transplantation were excluded from this study. Baseline characteristics were reported according to current guidelines [20–23].

### Transthoracic echocardiography

Certified personnel performed routine TTE using commercially available ultrasound systems (Philips iE33 or Epic, Philips Healthcare, Andover, Massachusetts, USA; GE Vivid 7 or E9 or E95, GE Healthcare, Milwaukee, Wisconsin, USA) in accordance with the recommendations of the American Society of Echocardiography (ASE) and the European Association of Echocardiography (now the European Association of Cardiovascular Imaging [EACVI]) [24, 25]. Left ventricular ejection fraction (LVEF) was calculated by Simpson’s biplane method [24, 25].

### Computed tomography

Pre-interventional CT examinations were performed using a 128-slice dual source CT system (Somatom Definition Flash, Siemens Healthcare, Forchheim, Germany). An intravascular injection of 45 mL of iodinated contrast media Iopromide (Ultravist 300, 300 mg/mL, Bayer Schering Pharma, Berlin, Germany) was administered at a flow rate of 5 mL/sec, followed by a second bolus of 35 mL at a flow rate of 2.5 mL/sec, and a subsequent 60 mL bolus of saline solution at the same flow rate. Bolus tracking was performed in the ascending aorta with a signal attenuation threshold of 100 Hounsfield units. A cranio-caudal scan direction was applied in all the protocols, with the scan extending from the lung apex to the pubic symphysis. The CT electrocardiography-gated high-pitch scan was automatically started based on the previous 10 heartbeats to reach the 60% RR interval at the level of the sinotubular junction.

### Quantification of RAC

Dedicated software (3mensio Vascular 8.1, PIE Medical Imaging BV, Maastricht, The Netherlands) was used for quantitative analysis of RAC [26, 27]. Within the left and right main renal artery, a centreline was drawn semi-automatically beginning at the ostium of the renal artery and proceeding to the distal renal artery in the region of the renal hilus. Within the proximal 20 mm of the respective renal artery, the calcification volume was segmented (Figure S1). The same procedure was applied to both left and right accessory renal arteries when present. The total volume of RAC (mm^3^) was defined (i) for main analysis as the sum of the left and right main renal arteries as well as the left and right accessory renal arteries, and (ii) for sensitivity analysis as the sum of the left and right main renal arteries only. Values of zero were reported for (i) main renal arteries that could not be measured due to nephrectomy or renal hypoplasia, and (ii) for non-existent accessory renal arteries due to anatomical variability. In the analyses, RAC was approached in two distinct ways: in the main analysis as a binary variable representing its presence or absence and in a supplementary analysis as a logarithmically transformed continuous variable for the subgroup of patients with RAC.

### Follow-up and endpoints

The date of echocardiography before TAVI (i.e., within 6 months prior to procedure) marked the date of study inclusion and the date of the TAVI procedure determined the start of follow-up. Cardiovascular death was defined as the primary endpoint. Death from unknown cause was considered as cardiovascular death [28]. Death from other causes was censored at the time of death in the main analysis and counted as a competing risk event in competing risk analysis. Patient records and/or phone calls were used to assess patient survival status.

### Statistical analyses

Continuous variables are reported as median and interquartile range [IQR] and categorical variables as absolute number and percentage. Normal distribution of variables was assessed visually. Univariate group comparisons were conducted using the Mann-Whitney-U test for continuous variables, the chi-square test or Fisher’s exact test for categorical variables as appropriate, and the specific chi-square test for ordinal variables. P-values in descriptive tables should be interpreted exploratively. The variable ‘ever smoker’ includes active smokers and ex-smokers who quit at least six months prior to inclusion.

A competing risk analysis was performed to accurately estimate the marginal probabilities of cardiovascular and non-cardiovascular deaths after TAVI, which represented the two competing event types in this study. Cumulative incidence curves were plotted and compared using a test analogous to the log-rank test for Kaplan-Meier curves [29]. Median follow-up time was estimated with the reverse Kaplan-Meier method, including all deaths as censored. All other analyses focused solely on the time to cardiovascular death. Time-to-event cardiovascular death analysis was done with Kaplan-Meier survival curves and log-rank tests. Time-dependent Cox regression modelling was performed using single and multiple covariates and resulting regression coefficients reported as hazard ratios (HR) with 95% confidence intervals. Proportional hazard assumptions were assessed for all variables using the scaled Schoenfeld residuals. Covariates for multivariable models were selected based on clinical relevance. Model fit was assessed using Harrell’s C-statistic and the Akaike information criterion (AIC). A sensitivity analysis was performed by including only the main renal arteries in Cox regression models. Statistical analyses were conducted using R version 4.2.0.

## Results

### Baseline characteristics

A total of 268 patients were included. Supplemental Table S1 presents baseline clinical characteristics. Median age was 83.6 years [IQR: 79.3–87.0], sex was equally distributed, and the majority exhibited one or more comorbidities. Diabetes and atrial fibrillation were more prevalent in the subgroup with RAC, while all the other clinical characteristics including cardiovascular risk factors, coronary artery disease (CAD), coronary artery bypass graft (CABG), and renal function pre- and post-TAVI, were similarly distributed between those with and without RAC. No notable difference was observed in the access site for TAVI between the subgroups. Echocardiographic indices of severe AS such as AVA were similar in both subgroups, with a slightly lower MTPG in those with RAC. LVEF was preserved in the majority of patients (median [IQR]: 58.0% [47.0–65.0]).

### Renal artery calcification

RAC was the main variable of interest in this study. Calcification was determined in both the left and right main renal arteries of 262 patients; calcification could not be measured in the left main renal artery of four and the right main renal artery of two patients. Fifty-five patients exhibited a left accessory renal artery, while 59 had a right accessory renal artery. Among the 268 patients included, 51 (19.0%) displayed a RAC value of zero indicating the absence of RAC. In the patients with RAC (*N* = 217 (81.0%)), the median total volume of RAC was 61.7 mm^3^ [25.5–130.8]. The left renal artery exhibited a median volume of 30.9 mm^3^ [9.4–78.6]) and the right renal artery a median volume of 25.3 mm^3^ [2.7–64.0]; Supplemental Table S2).

### Aortic valve and arterial calcification

Aortic valve calcification as reflected by an Agatston score value above zero was present in all patients, while left ventricular outflow tract (LVOT) and sinotubular calcification were present in the majority (81.0% and 56.3%, respectively) and ascending aorta calcification in almost half of the patients (47.0%; Supplemental Table S2). Calcification at other cardiovascular sites, including the aortic valve, LVOT, sinotubular junction, and ascending aorta, was prevalent in the study population regardless of the presence or absence of RAC (Supplemental Table S2). Aortic valve Agatston score and LVOT calcification were numerically lower in the subgroup with RAC, while moderate or severe calcification of the ascending aorta was found in 16.2% of patients with RAC versus 5.9% without (Supplemental Table S2).

### Survival after TAVI

**Fig. 1:**
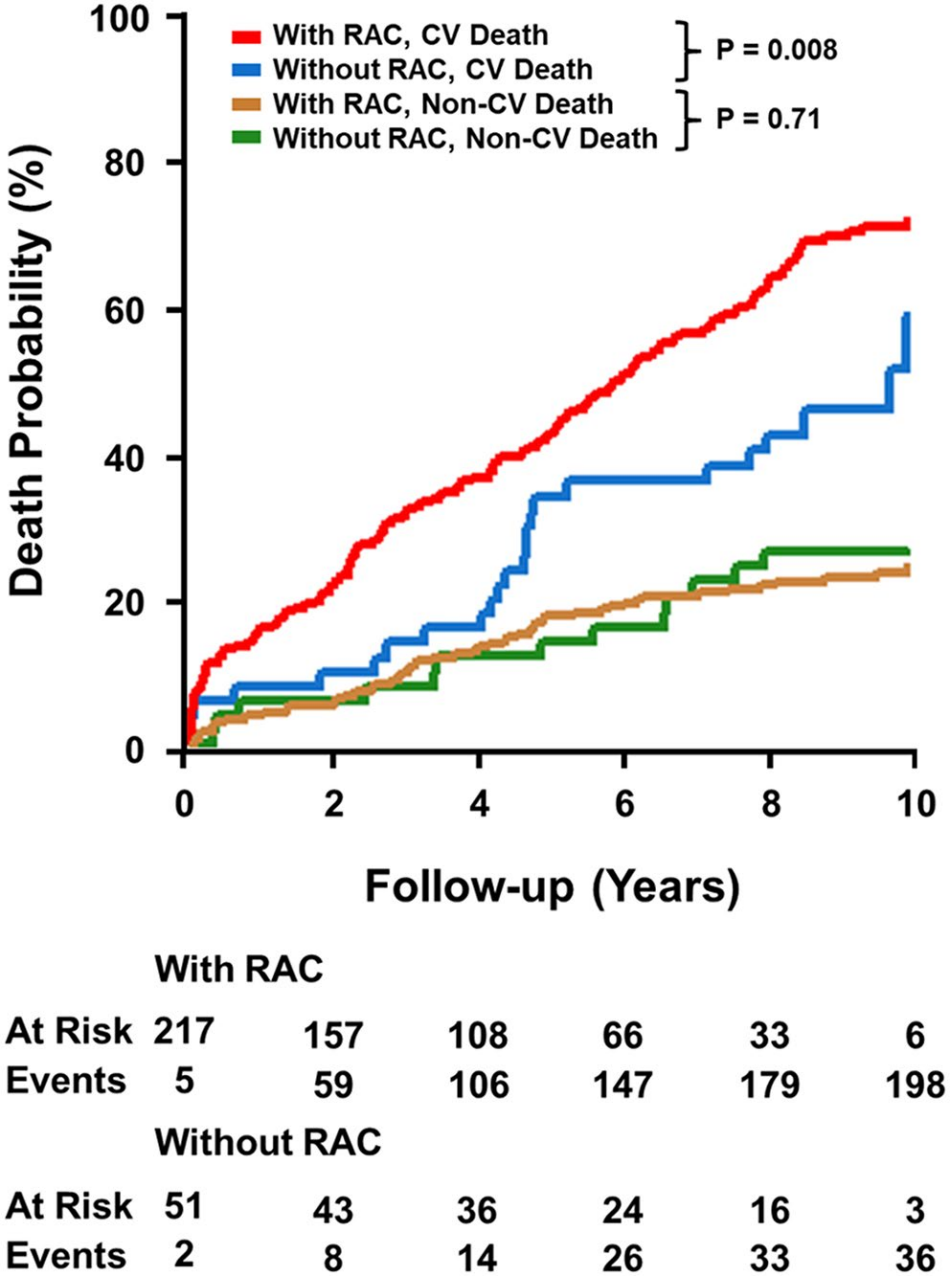
Cumulative incidence curves for competing risk analysis. Cumulative incidence curves for competing risk analysis of cardiovascular and non-cardiovascular death over a 10-year follow-up period, stratified by the presence or absence of RAC. Analogous log-rank test was used for subgroup comparisons. RAC, renal artery calcification; CV, cardiovascular

During a median follow-up time of 9.6 [8.6–10.7] years, 237 (88.4%) patients died, with 174 (73.4%) deaths occurring due to cardiovascular reasons. RAC was highly prevalent in patients suffering cardiovascular death (*N* = 150 (86.2%)). Stratified by the presence or absence of RAC as a binary variable, competing risk analysis of cardiovascular versus non-cardiovascular death with cumulative incidence curves revealed a higher occurrence of cardiovascular deaths in patients with RAC compared to those without RAC (analogous log-rank test P-value = 0.008; Fig. 1). In contrast, there was no evidence for a difference in non-cardiovascular deaths between the two binary RAC groups (analogous log-rank test P-value = 0.71; Fig. 1).

**Fig. 2:**
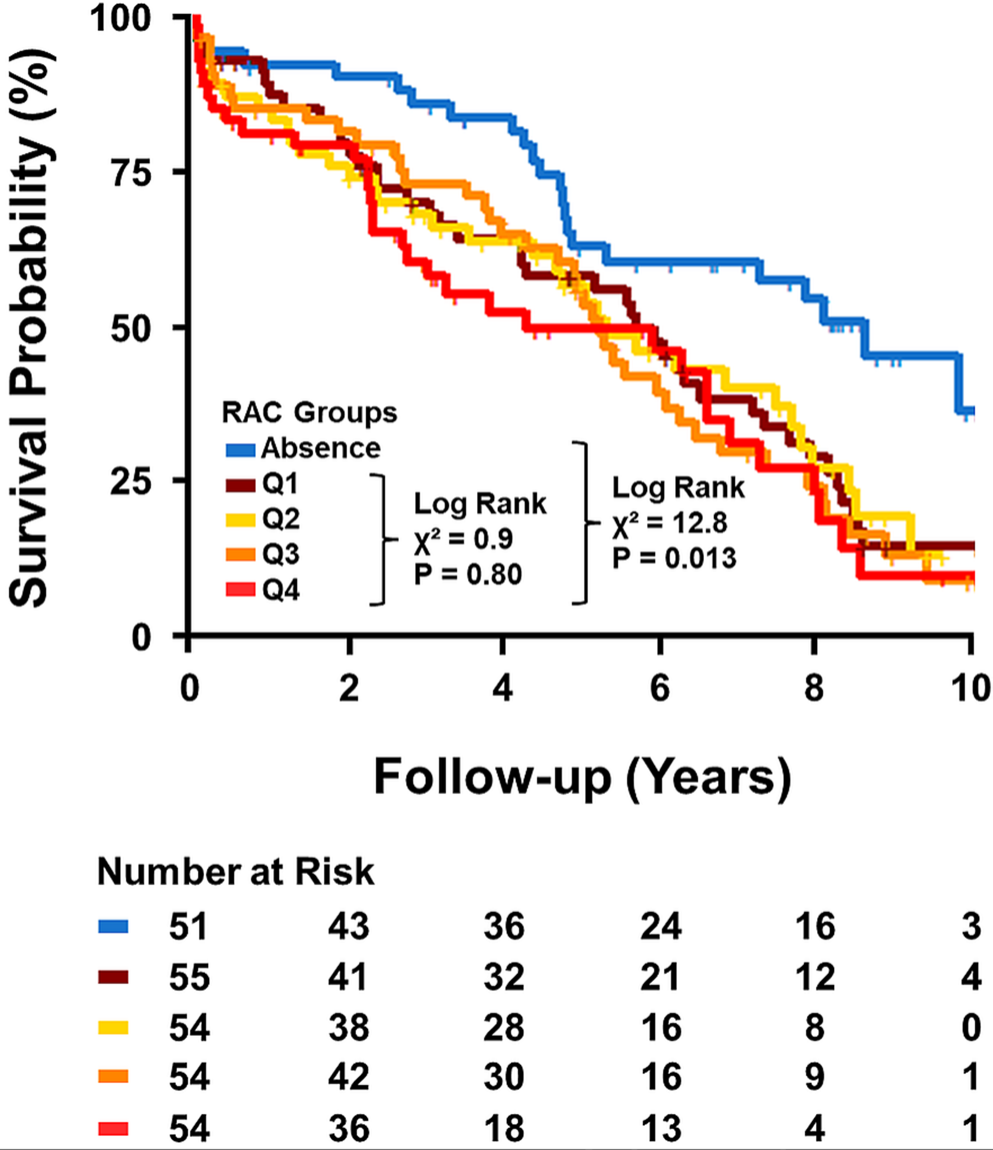
Survival curves for cardiovascular mortality according to subgroups of RAC. Kaplan-Meier survival probability for cardiovascular mortality over a 10-year follow-up period, stratified by subgroups of RAC. P-value calculated using log-rank test. RAC, renal artery calcification

Kaplan-Meier curves of time to cardiovascular death with and without RAC indicated a clear difference in survival (log-rank test P-value < 0.001; Supplemental Figure S2). Median survival times with 95% confidence bands were 5.2 [4.7–6.1] years for the group with RAC and 8.6 [4.8 – not reached] years for that without RAC, respectively. When RAC values above zero were subdivided into quartiles based on the amount of RAC, no difference in survival probability was observed between the quartiles (log-rank test P-value = 0.80; Fig. 2). In contrast, a difference between subgroups emerged when the patient group without RAC was included (log-rank test P-value = 0.013; Fig. 2).

Univariable Cox regression analysis revealed strong evidence for an association between the presence of RAC and a higher risk of cardiovascular death, with a hazard twice as high for patients with RAC (HR 2.05 [95% CI: 1.33–3.16]; P-value = 0.001; Supplemental Table S3). RAC as a continuous variable did not exhibit evidence for an association with the endpoint (HR 1.07 [0.93–1.23]; P-value = 0.35; data not shown). Similar results were observed for RAC values derived from the main renal arteries (HR 2.10 [1.36–3.24]; P-value < 0.001; Supplemental Table S3) and when analysed as a continuous variable (HR 1.06 [0.92–1.22]; P-value = 0.44; data not shown). The analysis also revealed an association between atrial fibrillation (HR 1.75 [1.28–2.40], P-value < 0.001] and creatinine levels measured before TAVI (pre-TAVI: HR 1.04 [1.02–1.06], P-value < 0.001; Supplemental Table S3) and 72 h after TAVI (post-TAVI: HR 1.03 [1.01–1.04], P-value < 0.0001; data not shown), both indicating an increased risk of cardiovascular death. While it was not possible to confirm a difference in RAC between patients with LVEF < 40% (*N* = 41 (15.3%)) and > 40% (*N* = 227 (84.7%)), LVEF < 40% was associated with cardiovascular death in univariable Cox regression analysis (P-value = 0.012; data not shown).

**Fig. 3:**
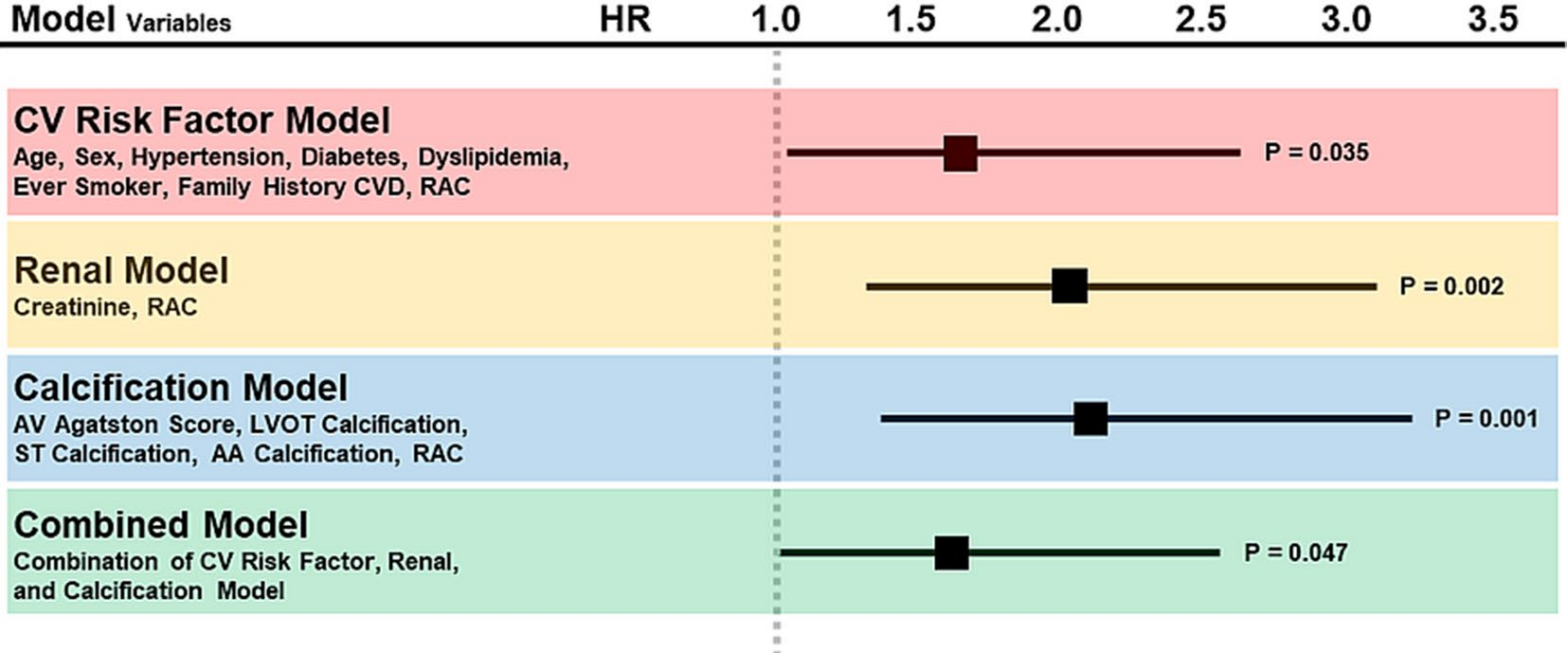
RAC hazard ratio for cardiovascular mortality in Cox regression models. Illustration of the hazard ratio for cardiovascular death associated with RAC in various Cox regression models. RAC, renal artery calcification; HR, hazard ratio

In multivariable Cox regression models, solid evidence persisted for the association between the presence of RAC and cardiovascular death (Fig. 3; Supplemental Table S4) independent of cardiovascular risk factors (Table 1, Model A), creatinine levels (Table 1, Model B), and calcification in other cardiovascular beds (Table 1, Model C). In the combined model considering clinical and cardiovascular risk factors, creatinine, and aortic valve calcification, patients with RAC exhibited a 1.6 times higher hazard of cardiovascular death compared to those without RAC (HR 1.61 [1.01–2.57]; P-value = 0.047;Fig. 3). Conversely, the absence of RAC reduced the risk of cardiovascular death by approximately 40% (HR 0.62 [0.38–0.99]). When atrial fibrillation was included in the combined model, RAC showed a slightly lower hazard of cardiovascular death compared to those without RAC (HR 1.51 [0.94–2.43]; P-value = 0.085; Table 2, Model A), but this reached significance when considering the main renal arteries (HR 1.66 [1.04–2.65]; P-value = 0.034; Supplemental Table S7, Model A).

**Table 1:** Multivariable Cox regression models for cardiovascular mortality

Variables	Cox Regression			Harrell’s C-statistic		Model Fit
	HR	95% CI	*P*-value	C-index	95% CI	AIC
A: Cardiovascular Risk Factor Model (*N* = 233, Events = 148)						
Age (per 1 year)	1.05	1.02–1.09	0.003	0.60	0.55–0.66	1361
Sex (male)	1.20	0.81–1.77	0.37			
Hypertension	1.48	0.93–2.34	0.10			
Diabetes	1.31	0.89–1.93	0.18			
Dyslipidaemia	1.13	0.79–1.60	0.51			
Ever smoker	1.56	1.04–2.35	0.031			
Family history CVD	0.89	0.63–1.25	0.49			
RAC	1.66	1.04–2.65	0.035			
B: Renal Model (*N* = 267, Events = 173)						
Creatinine (per 10 µmol/L)	1.03	1.02–1.05	< 0.001	0.61	0.56–0.66	1629
RAC	2.01	1.30–3.10	0.002			
C: Calcification Model (*N* = 265, Events = 173)						
Aortic valve Agatston score (per 100 units)	1.00	0.98–1.03	0.76	0.58	0.53–0.63	1636
LVOT calcification	0.62	0.42–0.92	0.018			
Sinotubular calcification	0.99	0.72–1.35	0.95			
Ascending aorta calcification	0.99	0.72–1.35	0.94			
RAC	2.07	1.34–3.21	0.001			

Association with cardiovascular mortality in multivariable Cox regression analysis. RAC was significantly associated with cardiovascular mortality, independent of clinical characteristics and cardiovascular risk factors (Model A), baseline creatinine levels (Model B), and calcification in other vascular beds (Model C). HR, hazard ratio; CI, confidence interval; AIC, Akaike information criterion. CVD, cardiovascular disease; RAC, renal artery calcification; LVOT left ventricular outflow tract

**Table 2:** Combined multivariable Cox regression models for cardiovascular mortality

Variables	Cox Regression				Harrell’s C-statistic		Model Fit
	HR		95% CI	*P*-value	C-index	95% CI	AIC
A: Combined Model (RAC binary variable) (*N* = 233, Events = 148)							
Age (per 1 year)	1.06		1.02–1.09	0.001	0.65	0.59–0.70	1349
Sex (male)	0.98		0.66–1.48	0.94			
Hypertension	1.33		0.83–2.11	0.23			
Diabetes	1.36		0.92–2.01	0.12			
Dyslipidaemia	1.12		0.78–1.61	0.54			
Ever smoker	1.71		1.12–2.60	0.013			
Family history CVD	0.91		0.64–1.28	0.58			
Atrial fibrillation	1.49		1.05–2.12	0.025			
Creatinine (per 10 µmol/L)	1.05		1.02–1.07	< 0.0001			
Aortic valve Agatston score (per 100 units)	1.00		0.97–1.02	0.87			
RAC	1.51		0.94–2.43	0.085			
B: Combined Model (RAC continuous variable) (*N* = 187, Events = 127)							
Age (per 1 year)	1.04		1.00–1.09	0.054	0.65	0.59–0.70	1103
Sex (male)	0.95		0.69–1.70	0.84			
Hypertension	1.32		0.84–2.33	0.29			
Diabetes	1.47		0.97–2.21	0.066			
Dyslipidaemia	0.99		0.67–1.49	0.95			
Ever smoker	1.44		0.89–2.35	0.15			
Family history CVD	0.95		0.62–1.32	0.81			
Atrial fibrillation	1.62		1.11–2.36	0.012			
Creatinine (per 10 µmol/L)	1.04		1.02–1.06	0.0001			
Aortic valve Agatston score (per 100 units)	1.00		0.97–1.02	0.93			
RAC	1.05		0.90–1.22	0.54			

Association with cardiovascular mortality was analysed using multivariable Cox regression, combining the Cardiovascular Risk Factor Model (Table 1, Model A), Renal Model (Table 1, )Model B, and Calcification Model (Table 1, )Model C, with adjustment for atrial fibrillation. RAC was included either as a binary variable (Model A) or as a continuous variable using logarithmic values for values above zero (Model B). HR, hazard ratio; CI, confidence interval; AIC, Akaike information criterion. CVD, cardiovascular disease; RAC, renal artery calcification; LVOT left ventricular outflow tract

The highest C-index and lowest AIC hence indicating the best model fit were observed for the combined model including major clinical and cardiovascular risk factors, atrial fibrillation, creatinine, aortic valve calcification, and RAC (Table 2, Model A). Since the main variable of interest was not normally distributed, a logarithmic transformation was performed on RAC (if present) as a continuous variable, which revealed no association with the endpoint in univariable (described above) and multivariable Cox regression models (HR 1.05 [0.90–1.22]; P-value = 0.54; Table 2, Model B). A sensitivity analysis for the main renal arteries yielded very similar results (Supplemental Table S5 – S7).

## Discussion

This study reveals that (1) RAC was highly prevalent among patients suffering cardiovascular death during long-term follow-up after TAVI; (2) RAC differentiated survivors from non-survivors in terms of cardiovascular but not non-cardiovascular death; (3) RAC independently predicted cardiovascular death after correction for age, sex, cardiovascular risk factors, impaired renal function, and aortic valve calcification; and (4) qualitative rather than quantitative assessment of RAC was important for outcome associations.

Degenerative AS is an age-related condition characterised by progressive calcification of the aortic valve [30, 31]. Although its pathogenesis may be similar to that of atherosclerosis, there are notable differences in the development of AS and atherosclerosis [32]. These distinctions may be attributed to the contribution of cardiovascular risk factors and the involvement of cardiovascular cells and their mediators [19, 33]. A minority of patients with severe AS does not exhibit any coronary artery stenosis or calcification indicating that degenerative changes of the cardiovascular system occur in a heterogeneous manner [34–36]. This is in line with observations in the current study population where the presence of RAC did not correspond well with calcification in other vascular beds or the aortic valve. While the reasons for this heterogeneity are incompletely understood [37], calcification of specific vascular beds may be utilised for event prediction independent of pathophysiological considerations. Calcification of the renal artery appears to be a particularly promising target in this context given its role as a biomarker for mortality in a normal population [15]. The current study underscores the utility of RAC for predicting cardiovascular events in patients with severe AS undergoing TAVI, evident through high hazard ratios for cardiovascular death as well as its independence from age, sex, major clinical and cardiovascular risk factors, impaired renal function, and aortic valve calcification.

Patients with severe AS routinely undergo a thoraco-abdominal CT examination before the TAVI procedure as part of pre-interventional planning [1, 8]. This allows for the evaluation of aortic valve calcification and characterisation of the access route [1, 8]. Thus, RAC can be easily identified on these routinely assessed images and then serve as an additional data point for event prediction. Although the renal artery can readily be evaluated by CT, the significance of RAC is poorly studied. To the best of our knowledge, there is only one published study investigating the impact of calcification in the renal arterial bed on mortality [15]. That study revealed that RAC was independently associated with a higher risk of all-cause mortality and provided incremental predictive value in a population of healthy out-patients without known cardiovascular disease regardless of cardiovascular risk factors and atherosclerosis [15]. While the endpoint of that research did not specifically focus on cardiovascular mortality, the study suggested that RAC is not merely a marker of systemic vascular calcium burden but may also offer additional prognostic information [15]. This interpretation is consistent with the findings of the current study, which explores the impact of RAC on predicting survival in patients with severe AS undergoing TAVI. A competing risk analysis indeed revealed a higher incidence of cardiovascular death in patients with RAC compared to those without, while no significant difference in non-cardiovascular death between the two groups was found. This observation does not stand in contrast to the published study in healthy out-patients since cardiovascular mortality was not assessed separately in that study [15]. Both observations highlight the clinical importance of an increased mortality risk associated with the presence of RAC during long-term follow-up across diverse populations.

Renal function is an established outcome predictor in various cardiovascular diseases including AS [19, 38]. The association between creatinine levels before and 72 h after TAVI and the risk of cardiovascular death demonstrated very strong evidence, albeit with a considerably smaller hazard ratio compared to RAC. Given that the association between RAC and the risk of cardiovascular death remained independent of major clinical parameters, cardiovascular risk factors, impaired renal function, and calcification in other vascular beds, and considering its stronger association compared to the aforementioned parameters, RAC may serve as a valuable prognostic parameter for post-TAVI follow-up. Conversely, the absence of RAC may reduce the risk of cardiovascular death significantly.

The risk of cardiovascular mortality was substantially attenuated when considering RAC as a continuous variable if present, suggesting that no discernible dose-dependent effect was observed in the current study population. This indicates that the qualitative assessment (i.e., presence or absence) of RAC is more pertinent than its quantitative analysis for evaluating the prognosis of patients after TAVI.

### Limitations

The retrospective single-centre design of this study is its main limitation. There may be a selection bias as the inclusion criteria required on-site CT. Kaplan-Meier curves illustrating time to cardiovascular death, although revealing a significant difference in survival, reflect unadjusted analyses and require careful interpretation. Nevertheless, the findings were largely supported by the adjusted analyses using multivariable Cox regression models. Measuring calcium volume in post-contrast images may lead to overestimation of calcification. It cannot be fully excluded that this inaccuracy contributes to the finding that only binary rather than logarithmic continuous calcium was a significant predictor of death. Large-scare prospective cohort studies on RAC in severe AS are required to validate the current findings.

## Conclusions

In patients with severe AS undergoing TAVI, RAC was highly prevalent among those suffering cardiovascular death during long-term follow-up. RAC clearly differentiated survivors from non-survivors in terms of cardiovascular but not non-cardiovascular death. RAC exhibited a strong association with cardiovascular death even after adjusting for age, sex, cardiovascular risk factors, impaired renal function, and calcification in other vascular beds. The qualitative assessment of RAC rather than its quantitative analysis proved to be important for determining outcome associations. Given its ready availability in pre-TAVI CT and favourable predictive properties, RAC may be considered for routine prognostic assessment of patients undergoing TAVI.

### Clinical perspectives

In patients with severe AS undergoing TAVI, thoraco-abdominal CT scans are conducted routinely for pre-interventional planning and can be employed for assessing RAC. Patients with RAC exhibit a high risk of cardiovascular death during follow-up, while those without RAC have an approximately 40% lower risk of cardiovascular death, independent of age, sex, cardiovascular risk factors, impaired renal function, and calcification in other vascular beds. Hence, measurement of RAC may serve as a valuable tool for predicting long-term survival of patients undergoing TAVI, offering the treating clinician an additional prognostic factor that can be used to enhance risk profiling and facilitate decision-making.

## Electronic supplementary material

Below is the link to the electronic supplementary material.

Supplementary Material 1

## Data Availability

The data supporting the findings of this study are included in the article or supplementary material and will be shared upon reasonable request to the corresponding author.

## Abbreviations

AS: Aortic Stenosis
AVA: Aortic Valve Area
CABG: Coronary Artery Bypass Graft
LV: Left Ventricle/Ventricular
LVEF: Left Ventricular Ejection Fraction
LVOT: Left Ventricular Outflow Tract
CT: Computed Tomography
MTPG: Mean Transaortic Pressure Gradient
PAD: Peripheral Artery Disease
RAC: Renal Artery Calcification
TAVI: Transcatheter Aortic Valve Implantation
TTE: Transthoracic Echocardiography
