# Supplementary material for: Pre-interventional renal artery calcification and survival after transcatheter aortic valve implantation
Source: Int J Cardiovasc Imaging. 2024 Dec 7;41(1):113–22. doi: 10.1007/s10554-024-03295-5 (PMC11742467; doi:10.1007/s10554-024-03295-5)
Supplement: Supplementary file 1 — Supplementary Material 1 [file 10554_2024_3295_MOESM1_ESM.docx]

**Supplemental Figure S1:** **Quantification of Renal Artery Calcification**


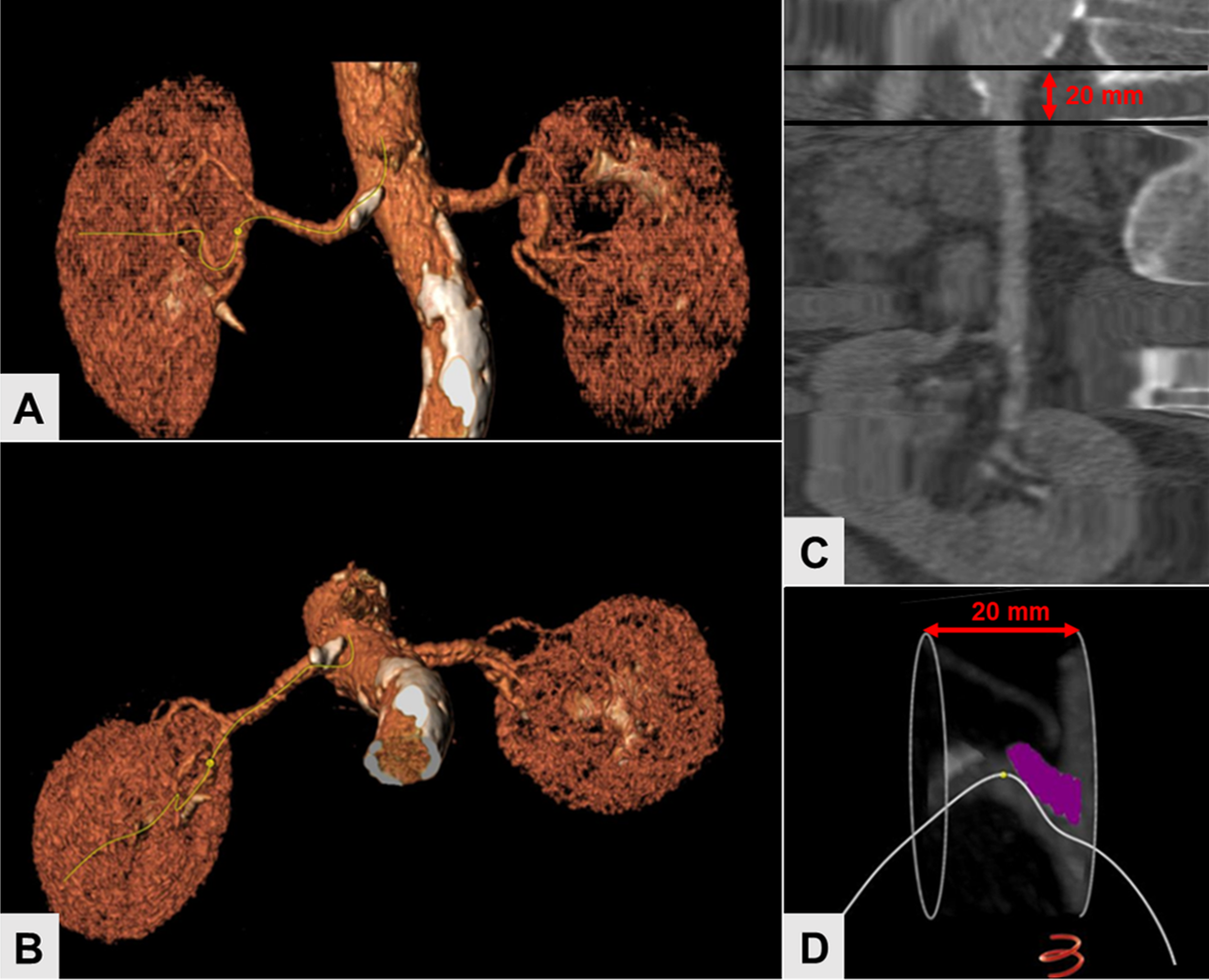


Panels A and B: Volume rendered transformation of aorta, renal arteries and kidneys with centreline depicted in the right renal artery. Panel C: Multiplanar reconstruction of the right renal artery along centreline, stretched view. Panel D: Maximum intensity projection of proximal segment of renal artery with colored segmentation of intravascular renal calcification.

**Supplemental Figure S2: Survival Curves for Cardiovascular Mortality According to Presence or Absence of RAC**


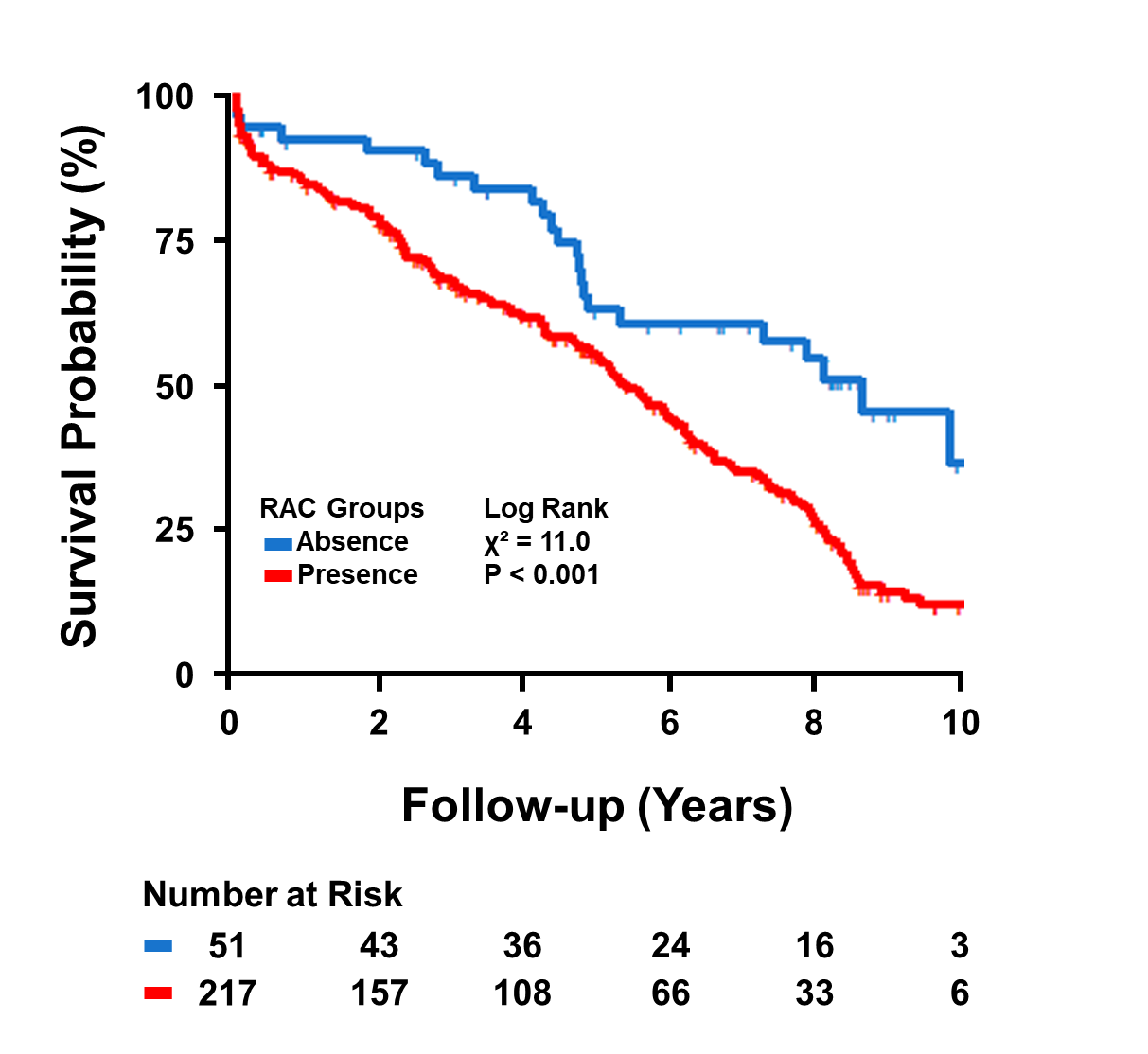


Kaplan-Meier survival probability for cardiovascular mortality over a 10-year follow-up period, stratified by the presence or absence RAC. P-value calculated using log-rank test. RAC, renal artery calcification.

**Supplemental Table S1: Baseline Clinical Characteristics**

| **Parameters** | **Overall (N = 268)** | **Without RAC (N = 51)** | **With RAC (N = 217)** | **P-value** |
| --- | --- | --- | --- | --- |
| **Age, years** | **83.6 [79.3 – 87.0]** | **82.4 [76.9 – 86.9]** | **84.0 [79.9 – 87.0]** | **0.22** |
| **Male, N (%)** | **133 (49.6)** | **24 (47.1)** | **109 (50.2)** | **0.80** |
| **BMI, kg/m^2^** | **26.1 [23.1 – 29.6]** | **26.1 [23.2 – 29.4]** | **26.1 [23.1 – 29.6]** | **0.78** |
| **Hypertension, N (%)** | **211 (78.7)** | **38 (74.5)** | **173 (79.7)** | **0.53** |
| **Diabetes, N (%)** | **63 (23.5)** | **7 (13.7)** | **56 (25.8)** | **0.10** |
| **Dyslipidaemia, N (%)** | **105 (39.2)** | **20 (39.2)** | **85 (39.2)** | **1.00** |
| **Atrial fibrillation, N (%)** | **90 (33.6)** | **9 (17.6)** | **81 (37.3)** | **0.012** |
| **Coronary artery disease, N (%)** | **145 (54.1)** | **28 (54.9)** | **117 (53.9)** | **1.00** |
| **CABG, N (%)** | **49 (18.3)** | **8 (15.7)** | **41 (18.9)** | **0.74** |
| **Peripheral artery disease, N (%)** | **44 (16.4)** | **6 (11.8)** | **38 (17.5)** | **0.43** |
| **Ever smoker, N (%)** | **112 (41.8)** | **25 (49.0)** | **87 (40.1)** | **0.34** |
| **Family history CVD, N (%)** | **124 (46.3)** | **26 (51.0)** | **98 (45.2)** | **0.80** |
| **EuroSCORE, %** | **4.7 [2.5 – 8.5]** | **4.5 [2.3 – 7.4]** | **4.8 [2.6 – 8.5]** | **0.35** |
| **Access site for TAVI, N (%)** |  |  |  | **0.90** |
| **Femoral** | **227 (84.7)** | **44 (86.3)** | **183 (84.3)** |  |
| **Non-femoral** | **41 (15.3)** | **7 (13.7)** | **34 (15.7)** |  |
| **Creatinine, µmol/L** | **95.0 [78.5 – 120.0]** | **96.0 [82.0 – 120.0]** | **94.5 [78.0 – 119.3]** | **0.73** |
| **Creatinine 72h post-TAVI, µmol/L** | **94.0 [74.0 – 123.5]** | **91.0 [74.0 – 111.0]** | **94.0 [74.0 – 124.8]** | **0.70** |
| **eGFR, mL/min/1.73m^2^** | **53.1 [39.9 – 69.3]** | **54.1 [45.2 – 65.4]** | **53.0 [39.0 – 70.3]** | **0.67** |
| **eGFR post-TAVI,** **mL/min/1.73m^2^** | **59.0 [44.0 – 72.0]** | **59.0 [49.3 – 71.8]** | **59.0 [43.0 – 71.5]** | **0.70** |
| **AKI at 30 days, stage 2 or 3, N (%)** | **4 (1.5)** | **0 (0.0)** | **4 (1.8)** | **0.74** |
| **AVA, cm^2^** | **0.7 [0.6 – 0.9]** | **0.7 [0.6 – 0.9]** | **0.7 [0.6 – 0.9]** | **0.72** |
| **MTPG, mmHg** | **44.0 [34.0 – 55.0]** | **50.0 [36.3 – 58.3]** | **43.0 [33.0 – 53.8]** | **0.038** |
| **LVEF, %** | **58.0 [47.0 – 65.0]** | **60.0 [50.0 – 66.0]** | **58.0 [45.0 – 64.0]** | **0.07** |

Values are given as median (IQR, interquartile range) for continuous variables or number (percentage) for categorical variables.

RAC, renal artery calcification; BMI, body mass index; CVD, cardiovascular disease; CABG, coronary artery bypass graft; EuroSCORE, European System for Cardiac Operative Risk Evaluation; eGFR, estimated glomerular filtration rate; TAVI, transcatheter aortic valve implantation**;** AKI, acute kidney injury; AVA, aortic valve area; MTPG, mean transaortic pressure gradient; LVEF, left ventricular ejection fraction.

**Supplemental Table S2: Baseline Calcification Characteristics**

| **Parameters** | **Overall (N = 268)** | **Without RAC (N = 51)** | **With RAC (N = 217)** | **P-value** |
| --- | --- | --- | --- | --- |
| **Renal artery calcification, volume (mm^3^)** |  |  |  |  |
| **Total** | **44.8 [8.4 – 109.9]** | **NA** | **61.7 [25.5 – 130.8]** | **NA** |
| **Left** | **21.0 [0.0 – 62.7]** | **NA** | **30.9 [9.4 – 78.6]** | **NA** |
| **Right** | **15.0 [0.0 – 49.7]** | **NA** | **25.3 [2.7 – 64.0]** | **NA** |
| **Logarithmic** | **4.1 [3.2 – 4.9]** | **NA** | **4.1 [3.2 – 4.9]** | **NA** |
| **Aortic valve Agatston score** | **813.7 [506.5 – 1299.1]** | **859.8 [584.8 – 1317.5]** | **812.0 [503.3 – 1298.2]** | **0.44** |
| **LVOT calcification, volume (mm^3^)** | **17.1 [1.3 – 114.0]** | **42.4 [4.8 – 114.0]** | **14.2 [1.3 – 113.2]** | **0.19** |
| **LVOT calcification, N (%)** | **217 (81.0)** | **43 (84.3)** | **174 (80.2)** | **0.77** |
| **Sinotubular calcification, N (%)** |  |  |  | **0.76** |
| **Absent** | **117 (43.7)** | **22 (43.1)** | **95 (43.8)** |  |
| **≤ 1/3** | **95 (35.4)** | **20 (39.2)** | **75 (34.6)** |  |
| **> 1/3 to ≤ 2/3** | **44 (16.4)** | **7 (13.7)** | **37 (17.1)** |  |
| **> 2/3** | **12 (4.5)** | **2 (3.9)** | **10 (4.6)** |  |
| **Ascending aorta calcification, N (%)** |  |  |  | **0.06** |
| **Absent** | **142 (53.0)** | **32 (62.7)** | **110 (50.7)** |  |
| **Mild** | **88 (32.8)** | **16 (31.4)** | **72 (33.2)** |  |
| **Moderate** | **22 (8.2)** | **1 (2.0)** | **21 (9.7)** |  |
| **Severe** | **16 (6.0)** | **2 (3.9)** | **14 (6.5)** |  |

Values are given as median (IQR, interquartile range) for continuous variables or number (percentage) for categorical variables.

RAC, renal artery calcification; LVOT, left ventricular outflow tract.

**Supplemental Table S3: Univariable Cox Regression Models for Cardiovascular Mortality**

| **Variables** | **Cox Regression** | | |  |
| --- | --- | --- | --- | --- |
|  | **HR** | **95% CI** | **P-value** |  |
| **Age (per 1 year)** | **1.03** | **1.01 – 1.05** | **0.015** |  |
| **Sex (male)** | **1.30** | **0.97 – 1.76** | **0.08** |  |
| **Hypertension** | **1.53** | **1.03 – 2.27** | **0.036** |  |
| **Diabetes** | **1.35** | **0.94 – 1.93** | **0.10** |  |
| **Dyslipidaemia** | **1.16** | **0.85 – 1.57** | **0.35** |  |
| **Atrial fibrillation** | **1.75** | **1.28 – 2.40** | **< 0.001** |  |
| **Coronary artery disease** | **1.30** | **0.96 – 1.76** | **0.09** |  |
| **Peripheral artery disease** | **1.56** | **1.07 – 2.27** | **0.022** |  |
| **Ever smoker** | **1.40** | **1.02 – 1.91** | **0.036** |  |
| **Family history CVD** | **1.00** | **0.73 – 1.37** | **0.98** |  |
| **Creatinine (per 10 μmol/L)** | **1.04** | **1.02 – 1.06** | **< 0.001** |  |
| **eGFR (per 10 mL/min/1.73m^2^)** | **0.92** | **0.85 – 1.00** | **0.04** |  |
| **RAC** | **2.05** | **1.33 – 3.16** | **0.001** |  |
| **RAC main renal arteries** | **2.10** | **1.36 – 3.24** | **< 0.001** |  |
| **Aortic valve Agatston score (per 100 units)** | **0.99** | **0.97 – 1.01** | **0.44** |  |
| **LVOT calcification** | **0.63** | **0.43 – 0.92** | **0.017** |  |
| **Sinotubular calcification** | **0.99** | **0.73 – 1.34** | **0.95** |  |

Association with cardiovascular mortality in univariable Cox regression analysis.

HR, hazard ratio; CI, confidence interval.

CVD, cardiovascular disease; eGFR, estimated glomerular filtration rate; RAC, renal artery calcification; LVOT, left ventricular outflow tract.

**Supplemental Table S4: Multivariable Cox Regression Models for Cardiovascular Mortality**

| **Variables** | **Cox Regression** | | |  | **Harrell’s C-statistic** | |  | **Model Fit** |
| --- | --- | --- | --- | --- | --- | --- | --- | --- |
|  | **HR** | **95% CI** | **P-value** |  | **C-index** | **95% CI** |  | **AIC** |
| **A (N = 268, Events = 174)** |  |  |  |  |  |  |  |  |
| **Age (per 1 year)**  **Sex (male)**  **RAC** | **1.04**  **1.47 1.87** | **1.01 – 1.07**  **1.07 – 2.02**  **1.21 – 2.88** | **0.010**  **0.018**  **0.005** |  | **0.59** | **0.54 – 0.64** |  | **1643** |
| **B (N = 267, Events = 173)** |  |  |  |  |  |  |  |  |
| **Age (per 1 year)**  **Sex (male)**  **Creatinine (per 10 μmol/L)**  **RAC** | **1.04**  **1.39 1.04**  **1.82** | **1.01 – 1.07**  **1.01 – 1.92**  **1.02 – 1.06 1.18 – 2.82** | **0.007**  **0.044**  **< 0.001**  **0.007** |  | **0.61** | **0.56 – 0.66** |  | **1624** |

Association with mortality in multivariable Cox regression analysis. RAC (binary) was significantly associated with cardiovascular mortality, independent of demographic characteristics (age and sex; Model A) and baseline creatinine levels (Model B).

HR, hazard ratio; CI, confidence interval; AIC, Akaike information criterion; RAC, renal artery calcification.

**Supplemental Table S5: Multivariable Cox Regression Models for Cardiovascular Mortality including RAC of Main Renal Arteries**

| **Variables** | **Cox Regression** | | |  | **Harrell’s C-statistic** | |  | **Model Fit** |
| --- | --- | --- | --- | --- | --- | --- | --- | --- |
|  | **HR** | **95% CI** | **P-value** |  | **C-index** | **95% CI** |  | **AIC** |
| **A (N = 268, Events = 174)** |  |  |  |  |  |  |  |  |
| **Age (per 1 year)**  **Sex (male)**  **RAC main renal arteries** | **1.04**  **1.46** | **1.01 – 1.07**  **1.06 – 2.01**  **1.24 – 2.95** | **0.010**  **0.020**  **0.003** |  | **0.59** | **0.54 – 0.64** |  | **1642** |
| **B (N = 267, Events = 173)** |  |  |  |  |  |  |  |  |
| **Age (per 1 year)**  **Sex (male)**  **Creatinine (per 10 μmol/L)**  **RAC main renal arteries** | **1.04**  **1.38**  **1.04**  **1.89** | **1.01 – 1.07**  **1.00 – 1.90**  **1.02 – 1.06**  **1.22 – 2.93** | **0.008**  **0.05**  **< 0.001**  **0.004** |  | **0.62** | **0.57 – 0.66** |  | **1623** |

Sensitivity analysis for RAC in the main renal arteries, excluding accessory renal arteries, using multivariable Cox regression. RAC main renal arteries (binary) was significantly associated with cardiovascular mortality, independent of demographic characteristics (age and sex; Model A) and baseline creatinine levels (Model B).

HR, hazard ratio; CI, confidence interval; AIC, Akaike information criterion; RAC, renal artery calcification.

**Supplemental Table S6: Multivariable Cox Regression Models for Cardiovascular Mortality including RAC of Main Renal Arteries**

| **Variables** | **Cox Regression** | | |  | **Harrell’s C-statistic** | |  | **Model Fit** |
| --- | --- | --- | --- | --- | --- | --- | --- | --- |
|  | **HR** | **95% CI** | **P-value** |  | **C-index** | **95% CI** |  | **AIC** |
| **A: Cardiovascular Risk Factor Model (N = 233, Events = 148)** | | | | | | | | |
| **Age (per 1 year)**  **Sex (male)**  **Hypertension**  **Diabetes**  **Dyslipidaemia**  **Ever smoker**  **Family history CVD**  **RAC main renal arteries** | **1.05 1.19 1.48**  **1.32 1.13**  **1.56 0.89**  **1.72** | **1.02 – 1.09 0.80 – 1.76**  **0.93 – 2.33 0.89 – 1.95 0.80 – 1.62**  **1.04 – 2.34 0.63 – 1.25**  **1.08 – 2.74** | **0.003**  **0.39**  **0.10**  **0.16**  **0.48**  **0.033**  **0.50**  **0.023** |  | **0.61** | **0.56 – 0.66** |  | **1360** |
| **B: Renal Model (N = 267, Events = 173)** | | |  |  |  |  |  |  |
| **Creatinine (per 10 μmol/L)**  **RAC main renal arteries** | **1.04**  **2.08** | **1.02 – 1.05**  **1.35 – 3.21** | **< 0.001**  **< 0.001** |  | **0.62** | **0.57 – 0.66** |  | **1628** |
| **C: Calcification Model (N = 265, Events = 173)** | | |  |  |  |  |  |  |
| **Aortic valve Agatston score (per 100 units)**  **LVOT calcification**  **Sinotubular calcification**  **Ascending aorta calcification**  **RAC main renal arteries** | **1.00 0.63**  **1.00 0.98**  **2.13** | **0.98 – 1.03 0.42 – 0.93**  **0.73 – 1.36 0.72 – 1.34**  **1.37 – 3.29** | **0.77**  **0.020**  **0.98**  **0.90**  **< 0.001** |  | **0.58** | **0.53 – 0.63** |  | **1635** |

Sensitivity analysis for RAC in the main renal arteries, excluding accessory renal arteries, using multivariable Cox regression. RAC main renal arteries (binary) was significantly associated with cardiovascular mortality, independent of clinical characteristics and cardiovascular risk factors (Model A), baseline creatinine levels (Model B), and calcification in other vascular beds (Model C).

HR, hazard ratio; CI, confidence interval; AIC, Akaike information criterion; CVD, cardiovascular disease; RAC, renal artery calcification; LVOT left ventricular outflow tract.

| **Variables** | **Cox Regression** | | |  | **Harrell’s C-statistic** | |  | **Model Fit** |
| --- | --- | --- | --- | --- | --- | --- | --- | --- |
|  | **HR** | **95% CI** | **P-value** |  | **C-index** | **95% CI** |  | **AIC** |
| **A: Combined Model (RAC binary variable) (N = 233, Events = 148)** | | | | | | | | |
| **Age (per 1 year)**  **Sex (male)**  **Hypertension**  **Diabetes**  **Dyslipidaemia**  **Ever smoker**  **Family history CVD**  **Atrial fibrillation**  **Creatinine (per 10 μmol/L)**  **Aortic valve Agatston score (per 100 units)**  **RAC main renal arteries** | **1.06**  **0.96**  **1.31**  **1.39**  **1.14**  **1.69**  **0.91**  **1.52**  **1.05**  **1.00**  **1.66** | **1.02 – 1.10 0.64 – 1.45**  **0.83 – 2.09 0.94 – 2.05**  **0.80 – 1.64**  **1.11 – 2.58**  **0.65 – 1.29**  **1.07 – 2.15**  **1.03 – 1.07**  **0.97 – 1.02**  **1.04 – 2.65** | **0.001**  **0.85**  **0.25**  **0.098**  **0.47**  **0.014**  **0.61**  **0.019**  **< 0.0001**  **0.83**  **0.034** |  | **0.64** | **0.59 – 0.69** |  | **1347** |
| **B: Combined Model (RAC continuous variable) (N = 186, Events = 127)** | | | |  |  |  |  |  |
| **Age (per 1 year)**  **Sex (male)**  **Hypertension**  **Diabetes**  **Dyslipidaemia**  **Ever smoker**  **Family history CVD**  **Atrial fibrillation**  **Creatinine (per 10 μmol/L)**  **Aortic valve Agatston score (per 100 units)**  **RAC main renal arteries** | **1.05**  **0.91**  **1.29**  **1.62**  **1.06**  **1.37**  **0.97**  **1.74**  **1.05**  **1.00**  **1.00** | **1.00 – 1.09**  **0.57 – 1.44**  **0.77 – 2.16**  **1.07 – 2.46**  **0.71 – 1.59**  **0.83 – 2.25**  **0.67 – 1.42**  **1.19 – 2.54**  **1.02 – 1.07**  **0.97 – 1.02**  **0.86 – 1.17** | **0.035**  **0.68**  **0.33**  **0.024**  **0.78**  **0.22**  **0.89**  **0.004**  **< 0.0001**  **0.77**  **0.96** |  | **0.64** | **0.58 – 0.70** |  | **1098** |

**Supplemental Table S7: Combined Multivariable Cox Regression Models for Cardiovascular Mortality including RAC of Main Renal Arteries**

Sensitivity analysis for RAC in the main renal arteries, excluding accessory renal arteries, using multivariable Cox regression. RAC main renal arteries (binary) was significantly associated with cardiovascular mortality, independent of clinical characteristics including atrial fibrillation and cardiovascular risk factors, after adjusting the models for baseline creatinine levels and aortic valve calcification in a combined model (Model A). There was no association of outcome with logarithmic values of RAC in the main renal arteries above zero (Model B).

HR, hazard ratio; CI, confidence interval; AIC, Akaike information criterion; CVD, cardiovascular disease; RAC, renal artery calcification; LVOT left ventricular outflow tract.
